# The impact of a summative national prescribing assessment and curriculum type on the development of the prescribing competence of junior doctors

**DOI:** 10.1007/s00228-023-03567-4

**Published:** 2023-09-22

**Authors:** Erik M. Donker, Hayaudin Osmani, David J. Brinkman, Floor van Rosse, Ben Janssen, Wilma Knol, Glenn Dumont, Philippe G. Jorens, Alain Dupont, Thierry Christiaens, Jeroen van Smeden, Itte de Waard-Siebinga, Laura E. J. Peeters, Ronald Goorden, Marleen Hessel, Birgit I. Lissenberg-Witte, Milan C. Richir, Michiel A. van Agtmael, Cornelis Kramers, Jelle Tichelaar, on behalf of the Education committee of the Dutch Society for Clinical Pharmacology and Biopharmacy

**Affiliations:** 1 Unit Pharmacotherapy, Department of Internal Medicine, Amsterdam UMC, Location VUmc, De Boelelaan 1117, 1081HV Amsterdam, The Netherlands; 2 Research and Expertise Centre in Pharmacotherapy Education (RECIPE), Amsterdam, The Netherlands; 3 Department of Hospital Pharmacy, Erasmus MC, University Medical Center Rotterdam, Rotterdam, The Netherlands; 4 Department of Pharmacology and Toxicology, Maastricht University, Maastricht, The Netherlands; 5 Department of Geriatric Medicine, University Medical Center Utrecht, Utrecht University, Utrecht, The Netherlands; 6 Department of Hospital Pharmacy and Clinical Pharmacology, Amsterdam UMC, Location AMC, Amsterdam, The Netherlands; 7 Department Pharmacotherapy, Antwerp University Hospital, University of Antwerp, Antwerp, Belgium; 8 Department of Clinical Pharmacology, Free University of Brussels (VUB), Brussels, Belgium; 9 Clinical Pharmacology, Department of Basic and Applied Medical Sciences, Ghent University, Ghent, Belgium; 10 Department of Education, Centre for Human Drug Research, Leiden, The Netherlands; 11 Department of Clinical Pharmacy and Toxicology, Leiden University Medical Center, Leiden, The Netherlands; 12 Department Clinical Pharmacy and Pharmacology, University Medical Center Groningen, Groningen, The Netherlands; 13 Department of Internal Medicine, Erasmus MC, University Medical Center Rotterdam, Rotterdam, The Netherlands; 14 Radboud University Medical Center, Nijmegen, The Netherlands; 15 Department of Epidemiology and Data Science, Amsterdam UMC, Location VUmc, Amsterdam, The Netherlands; 16 Department of Surgery, University Medical Center Utrecht, Utrecht, The Netherlands; 17 Pharmacology-Toxicology and Internal Medicine, Radboud University Medical Center, Nijmegen, The Netherlands

**Keywords:** Clinical pharmacology, Education, Assessment, Pharmacotherapy

## Abstract

**Purpose:**

The primary aim of this study was to investigate the effect of including the Dutch National Pharmacotherapy Assessment (DNPA) in the medical curriculum on the level and development of prescribing knowledge and skills of junior doctors. The secondary aim was to evaluate the relationship between the curriculum type and the prescribing competence of junior doctors.

**Methods:**

We re-analysed the data of a longitudinal study conducted in 2016 involving recently graduated junior doctors from 11 medical schools across the Netherlands and Belgium. Participants completed three assessments during the first year after graduation (around graduation (+ / − 4 weeks), and 6 months, and 1 year after graduation), each of which contained 35 multiple choice questions (MCQs) assessing knowledge and three clinical case scenarios assessing skills. Only one medical school used the DNPA in its medical curriculum; the other medical schools used conventional means to assess prescribing knowledge and skills. Five medical schools were classified as providing solely theoretical clinical pharmacology and therapeutics (CPT) education; the others provided both theoretical and practical CPT education (mixed curriculum).

**Results:**

Of the 1584 invited junior doctors, 556 (35.1%) participated, 326 (58.6%) completed the MCQs and 325 (58.5%) the clinical case scenarios in all three assessments. Junior doctors whose medical curriculum included the DNPA had higher knowledge scores than other junior doctors (76.7% [SD 12.5] vs. 67.8% [SD 12.6], 81.8% [SD 11.1] vs. 76.1% [SD 11.1], 77.0% [12.1] vs. 70.6% [SD 14.0], *p* < 0.05 for all three assessments, respectively). There was no difference in skills scores at the moment of graduation (*p* = 0.110), but after 6 and 12 months junior doctors whose medical curriculum included the DNPA had higher skills scores (both *p* < 0.001). Junior doctors educated with a mixed curriculum had significantly higher scores for both knowledge and skills than did junior doctors educated with a theoretical curriculum (*p* < 0.05 in all assessments).

**Conclusion:**

Our findings suggest that the inclusion of the knowledge focused DNPA in the medical curriculum improves the prescribing knowledge, but not the skills, of junior doctors at the moment of graduation. However, after 6 and 12 months, both the knowledge and skills were higher in the junior doctors whose medical curriculum included the DNPA. A curriculum that provides both theoretical and practical education seems to improve both prescribing knowledge and skills relative to a solely theoretical curriculum.

**Supplementary Information:**

The online version contains supplementary material available at 10.1007/s00228-023-03567-4.

## Introduction

A substantial proportion of European medical students lack adequate prescribing knowledge and skills at graduation, probably because, among other aspects, they had too little education in clinical pharmacology and therapeutics (CPT) during their undergraduate training [1, 2]. It is often assumed that the prescribing competence of these students will improve once they become junior doctors, as they gain clinical experience. However, recently, we showed that the prescribing knowledge and skills of junior doctors (recent graduates) in the Netherlands and Flanders (Belgium) did not improve during the first year after graduation [3]. This is troubling because most hospital prescriptions (63–78%) are written out by junior doctors, who make the most prescribing errors (9–10% of all their prescriptions) [4–6]. This poor prescribing unfavourably affects patient safety, treatment effectiveness, and healthcare costs [4, 7, 8].

Adequate prescribing competence of graduating medical students stands high on the agenda of (inter)national societies, such as the European Association for Clinical Pharmacology and Therapeutics (EACPT) and the Dutch Society for Clinical Pharmacology and Biopharmacy [9, 10]. A final assessment of prescribing competence could be a first step to ensure that medical students have acquired sufficient prescribing knowledge and skills before graduation. Moreover, utilizing such an assessment could guide the teaching and training in clinical pharmacology and therapeutics (CPT) into the desired direction [11, 12]. The European Prescribing Exam (EuroPE^+^) was developed for this purpose and has been distributed and used among European medical schools [13]. In addition, the Prescribing Safety Assessment (PSA) has been developed in the UK and the Dutch National Pharmacotherapy Assessment (DNPA) in the Netherlands [10, 14–16]. The DNPA, developed in 2014 by the Dutch Society for Clinical Pharmacology and Biopharmacy, consists of 60 multiple choice questions (MCQ) focusing on prescribing safety such as ready knowledge (e.g. the mechanisms of action, clinically relevant side-effects, and contraindications) about the drugs responsible for the majority of medication-related harm and hospital admissions. [10, 16]. However, it is currently not known what the effect of a national prescribing safety examination is on the level and development of prescribing knowledge and skills of junior doctors in the year after graduation.

Besides a final prescribing safety assessment, another way to improve CPT education is to provide a combination of theoretical (e.g. lectures, seminars, self-study, written exams) and practical (e.g. clinics, bedside teaching, prescribing for real patients) teaching in medical curricula. Studies have shown that medical students who have followed problem-based learning, which is most probably more embedded in practical learning, have better prescribing knowledge and skills [17–19]. Moreover, enriching the learning context with real patients has been shown to improve students’ prescription-writing skills [20]. At the moment, all Dutch and Flanders (Belgium) medical schools have mixed learning curricula. However, little is known about the effect of different CPT curricula on the prescribing competence of junior doctors.

The main aim of this study was to investigate the effect of the Dutch National Pharmacotherapy Assessment as part of the medical curriculum on the level and development of prescribing knowledge and skills of junior doctors. The second aim was to evaluate the relationship between the curriculum type and the prescribing competence of junior doctors. We hypothesised that the knowledge-based DNPA would improve the knowledge but not the skills of junior doctors, and that both theoretical and practical teaching during undergraduate education delivers both more knowledgeable and skilful junior doctors.

## Methods

### Study design and participants

This study is a sub-analysis of data from a longitudinal prospective cohort study assessing the knowledge and skills of recently graduated junior doctors from 11 medical schools in the Netherlands (*n* = 8) and Belgium (*n* = 3) during three moments in their first year after graduation: around (+ / − 4 weeks) graduation (assessment 1), 6 months after graduation (assessment 2), and 1 year after graduation (assessment 3) [3]. In total, all 1584 graduating medical students (July 2016–March 2017) were invited to participate in this study. During this period, one medical school implemented the DNPA as a summative examination during the 5th year of its undergraduate curriculum, whereas the other medical schools (numbered 1–10) used other assessments (e.g. pharmacotherapy questions integrated in large medical exams) only. To establish the type of curriculum of the medical schools in the period before 2016, we used raw data from a previously published study [21]. Curricula are classified as ‘theoretical’ when CPT is taught by means of lectures, self-study, and working groups, whereas curricula are classified as practical when CPT education is provided during clinics, with bedside teaching and prescribing for real patients. Integration of both types of teaching is classified as a ‘mixed’ curriculum.

Permission for the study in the participating medical schools was granted by the Ethics Review Board of the Netherlands Association of Medical Education (NVMO-ERB 729). The study was funded by ZonMw (The Dutch Organisation for Health Research and Development), project no. 83600095004. Participants provided written informed consent prior to participation and were compensated with a 50-euro voucher for their time.

### Design, validity, and reliability of assessment tool

The assessment tool has been described in detail elsewhere [3]. In short, each assessment contained 35 multiple choice questions (MCQs) to assess prescribing knowledge, focusing on medication safety (i.e. factual drug knowledge such as contraindications, and interactions). These questions were extracted from the DNPA database [10, 16] and were different from the questions used in the summative examination of the junior doctors who graduated from school X. The MCQs were divided into seven different topics: (1) analgesics, (2) anticoagulants, (3) antibiotics, (4) cardiovascular drugs, (5) antidiabetics, (6) psychotropics, (7) basic pharmacokinetics and drug calculations. Furthermore, to assess the prescribing skills (i.e. rational prescribing), each assessment contained three clinical polypharmacy case scenarios (about renal impairment, anticoagulants, and pain management), set up by a group of clinically active senior clinical pharmacologists and medical specialists (e.g. internist, surgeon, and general practitioner) from all participating medical schools. Each case required its own treatment plan, including a (non)pharmacological policy and follow-up management. In general, each case required two main additions/alterations (e.g. starting pain treatment and changing a medication because of a clinically relevant drug-drug interaction interaction) and one to three minor alterations (e.g. correcting the timing of drug administration).

The MCQs and the clinical case scenarios had high content validity, with 75.8% and 72.7% of all knowledge and skills questions being rated as ‘essential’, respectively, by clinical pharmacologists not involved in this study [3, 16]. Reliability tests showed sufficient internal consistency for all three assessments (Cronbach alpha of 0.70, 0.69, and 0.76, respectively) and poor to adequate ability to distinguish good from poor students (*R*_ir_-scores; range − 0.02–0.46). The latter is inseparable with examining ready knowledge (i.e. easily accessible information for immediate use or application). The MCQs of assessment 2 seemed to be easier than those of assessments 1 and 3, as became apparent with a control group [3].

### Data collection

A local coordinator was appointed at each medical school to invite all (nearly) graduated students to participate in this study. The assessments were online, remote without surveillance, and each took about 60 min to complete (but there was no time limit). When necessary, reminders were sent out after 1 or 2 weeks. For the knowledge part, no external resources such as formularies were allowed, whereas for the skills part, this was allowed. Third parties were not allowed to be consulted. The collected data of all participants was encrypted and anonymized.

### Scoring and data analysis

For the knowledge part, the MCQs were marked as either correct or incorrect. Scores are given as a percentage of the maximum correct score. Since the examination assesses ready knowledge and students are expected to perform well on this, we used a pass grade of 85% correct answers, as used by the DNPA and EuroPE^+^ [10, 16].

For the skills part, the clinical cases were independently scored by two investigators (ED (clinical pharmacologist in training) and DB (clinical pharmacologist)), blinded for participant information, using an answer grading rubric based on national guidelines [3]. In the case of discrepancy, the expert group involved in creating the questions was consulted to reach consensus. Three aspects were scored: pharmacological and non-pharmacological policy, and follow-up management. These were scored either insufficient (0 points), sufficient (1 point), or good (2 points). The total score determined the classification of the treatment plan: insufficient (0–1 points), sufficient (2–3 points), or good (4–6 points).

For all longitudinal data, we used linear mixed models (continuous data) or generalized estimating equations (ordinal data) to assess the differences in scores. To assess the differences within one assessment, we performed either chi-square tests, independent *T*-tests, or ANOVA tests (with post-hoc Tukey HSD). Analysis was performed in SPSS 26.0 (IBM Corp., Armonk, NY, USA), and in Stata version 14 (StataCorp LLC, 2020).

## Results

Of the 1584 invited junior doctors, 556 (35.1%) participated, 326 (58.6%) completed the MCQs, and 325 (58.5%) the clinical case scenarios in all three assessments. On the basis of the prespecified criteria, five curricula were classified as theoretical and six as mixed. Demographic information is given in Table 1.

**Table 1:** Demographics

**Medical school**	**DNPA**	**Type of curriculum**	**Type of assessments in curriculum**	**No. of participants**	**Percentage (%)**
X	Yes	Mixed	Written, oral	57	17.5
1	No	Mixed	Written, portfolio, in clinics	10	3.1
2	No	Mixed	Written, oral, portfolio, OSCE, in clinics	34	10.4
3	No	Mixed	Written, in clinics	30	9.2
4	No	Mixed	Written, oral, portfolio	22	6.7
5	No	Mixed	Written, oral, in clinics	23	7.1
6	No	Theoretical	Written, OSCE	45	13.8
7	No	Theoretical	Written	47	14.4
8	No	Theoretical	Written	17	5.2
9	No	Theoretical	Written, oral, in clinics	31	9.5
10	No	Theoretical	Written, portfolio	10	3.1
				326	100

*DNPA *Dutch National Pharmacotherapy Assessment, *OSCE *objective structured clinical examination

### Knowledge

**Fig. 1:**
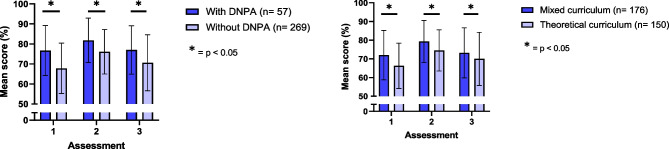
Mean knowledge score of junior doctors who graduated from medical schools that did or did not include the Dutch National Pharmacotherapy Assessment in the medical curriculum and a mixed or theoretical curriculum. Error bars show the standard deviation

Junior doctors with the DNPA in their curriculum outperformed other junior doctors (76.7 ± 12.5% vs. 67.8 ± 12.6%, 81.8 ± 11.1% vs. 76.1 ± 11.1%, 77.0 ± 12.1% vs. 70.6 ± 14.0%, *p* < 0.05 for all three assessments, respectively)(Fig. 1 and Supplementary Table 1). Also, junior doctors taught with a mixed curriculum outperformed junior doctors taught with a theoretical curriculum (72.0 ± 13.2% vs. 66.3 ± 12.1%, 79.3 ± 11.2% vs. 74.5 ± 11.0%, 73.2 ± 13.4% vs. 70.0 ± 14.2%, *p* < 0.05 for all three assessments respectively) (Fig. 1 and Supplementary Table 1). On average, junior doctors who had taken the DNPA during their medical education and junior doctors taught with a mixed curriculum were more likely to pass the assessments than the other junior doctors (36.8% vs. 17.6% and 26.3% vs. 14.7%, respectively) (Supplementary Table 2). Nonetheless, the average score of all subgroups per assessment was below the predefined pass grade of 85%. The comparison between school X and the individual medical schools can be found in Supplementary Table 3 and Supplementary Fig. 1.

In general, linear mixed modelling revealed no significant differences in the development of knowledge scores between the junior doctors who graduated with or without the DNPA in their curriculum, or between the junior doctors taught with a theoretical or mixed curriculum (*p* = 0.10 and *p* = 0.11, respectively). However, for the specific assessments, junior doctors taught with a theoretical curriculum showed a greater improvement in prescribing knowledge after 1 year (increase of 3.7% from baseline) than junior doctors taught with a mixed type curriculum (increase of 1.2% from baseline, *p* = 0.038).

### Skills

**Fig. 2:**
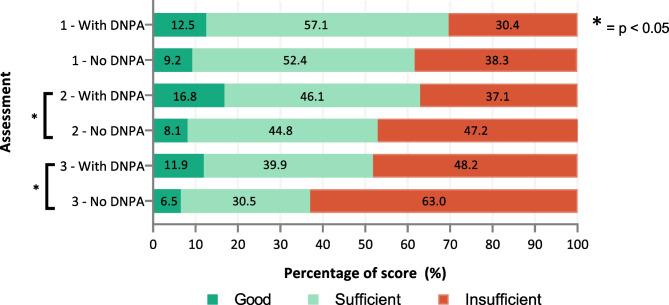
Total skills score of junior doctors who graduated from medical schools that did or did not include the Dutch National Pharmacotherapy Assessment in the medical curriculum

**Fig. 3:**
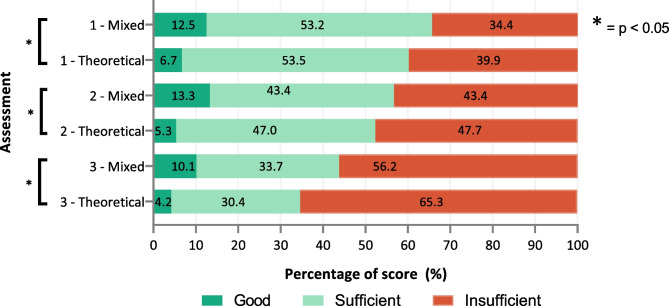
Total skills score of junior doctors who graduated from medical schools with a mixed or theoretical curriculum

At graduation (assessment 1), there was no difference in skills scores between the junior doctors with the DNPA in their curriculum and the other junior doctors (*p* = 0.110), but in assessments 2 and 3, the junior doctors with the DNPA in their curriculum had significantly higher scores (*p* = 0.001 for both) (Fig. 2). Junior doctors taught with a mixed curriculum outperformed junior doctors taught with a theoretical curriculum in all three assessments (*p* < 0.05 in all assessments) (Fig. 3). This could mainly be ascribed to a difference in the number of treatments plans scored as ‘good’. Nonetheless, skills deteriorated after graduation, regardless of where junior doctors graduated. The comparison between all individual medical schools can be found in Supplementary Fig. 2.

Generalized estimating equations showed that, over time, there was less deterioration in prescribing skills in the junior doctors with the DNPA in their curriculum and in the junior doctors taught with a mixed curriculum compared with the other junior doctors (both *p* < 0.001).

## Discussion

This study suggests that implementing the Dutch National Pharmacotherapy Assessment improves the prescribing knowledge but not necessarily the prescribing skills of junior doctors at graduation. Moreover, including practical CPT education in the medical curriculum is associated with more knowledgeable and skilful junior doctors. Nevertheless, the average prescribing knowledge and skills of the junior doctors involved in our study was below the predefined level of 85% correctness and did not improve in the year after graduation.

Assessment is an important component of a medical curriculum. The aim of assessment is, among other reasons, to evaluate whether students meet pre-determined learning objectives. A plausible reason why the DNPA only led to better prescribing knowledge and not to better prescribing skills at graduation is that the DNPA contains questions that assess knowledge-level learning objectives. In this study, we also assessed skills-level learning objectives by evaluating junior doctors’ capability to set up treatment plans for clinical polypharmacy case scenarios. Skills are typically learned during practical education and, indeed, our study shows that a curriculum that includes practical education leads to better prescribing knowledge and skills. However, in assessments two and three, junior doctors who had taken the DNPA during their training outperformed others. This suggests that the DNPA improves prescribing knowledge which is essential for enhancing prescribing skills in clinical practice. This hypothesis is supported by the finding in our study that junior doctors with the DNPA in their curriculum mainly performed better than juniors taught solely with theoretical education (Supplementary Fig. 2). Of course, it could be questioned whether the differences in prescribing knowledge are clinically relevant. However, on average, junior doctors who had taken the DNPA during their medical education and junior doctors taught with a mixed curriculum were more likely to pass the assessments than the other junior doctors. Despite the improved prescribing knowledge of junior doctors who had taken the DNPA during training and the improved prescribing knowledge and skills of junior doctors who had followed more practical education, the overall prescribing competence of the participants was still insufficient. Therefore, as recommended by the Joint British Pharmacological Society and EACPT (BPS/EACPT) Working Group on Safe Prescribing in 2007 and later by EACPT Education Working Group in 2018, both undergraduate and postgraduate education and training in CPT must be intensified, modernized, and harmonized [22, 23].

To assess whether future doctors have acquired sufficient prescribing skills, national prescribing safety assessments could incorporate more skills-type questions in their exams, such as prescribing for clinical case scenarios or performing medication reviews. The Prescribing Safety Assessment (PSA) in the UK and the European Prescribing Exam (EuroPE^+^) already have such skills questions [13–15], but it is not known whether this leads to more skilful junior doctors after being graduated.

The PSA is mandatory in most UK medical schools, and Foundation Year 1 doctors are required the pass the exam in order to progress to year 2. The EuroPE^+^ is currently being piloted as a formative assessment in twelve European medical schools, with the aim to incorporate the examination in all European medical schools as a summative exam for all penultimate or final-year medical students [13]. Currently, the DNPA is used as a summative assessment by seven medical schools in the Netherlands and as a formative assessment in one medical school. It is not known which manner of assessment is more effective. This question is becoming increasingly relevant as several medical schools in the Netherlands are switching to ‘programmatic assessment’. In ‘programmatic assessment’ curricula, information about the student's learning process is continuously collected via the so-called data points (e.g. feedback, exams, objective structured clinical examination (OSCE), et cetera) [24]. This allows teachers to monitor and adjust the student’s learning process. An important difference with conventional curricula is that not the individual exam, but the collection of data points over a longer period is assessed ‘summatively’ by an assessment committee [24]. One could argue that assessing prescribing knowledge and skills is of such importance that it should always be graded by summative assessment. On the other hand, formative assessments are more appropriate to drive learning [25–27]. A study comparing results between medical schools with summative or formative assessments might resolve this question.

There are possible reasons why junior doctors who had the DPNA in their medical curriculum performed better than other junior doctors in terms of prescribing knowledge but not prescribing skills. First, the DNPA was used in only one medical school, and it is possible that this school had a more effectively integrated CPT curriculum and teaching programme (constructive alignment) than the other medical schools. Second, there were only 57 junior doctors from this medical school compared with 269 junior doctors from the other medical schools, which may lead to differences in demographics. In our earlier study involving the same cohort, we found that non-surgical junior doctors outperformed surgical doctors [3]. However, there were similar proportions of surgical and non-surgical junior doctors among graduates from medical school X and the other medical schools, but fewer research physicians (3.5% vs. 11.5%, Supplementary Tables 4 and 5). We previously found that physician-researchers underperformed in the knowledge part compared with non-registrars and registrars in assessments 2 and 3 [3].

Our study also confirms that the prescribing knowledge and skills of junior doctors are insufficient since the majority of the participants did not pass the assessments [28–30]. As discussed elsewhere, the majority of the junior doctors worked in clinical practice (86.4%) and thus the assessed topics should be familiar and well-known [3]. We believe, like the BPS/EACPT Working Group on Safe Prescribing and Jansen et al. [16, 23], that all junior doctors should have broad knowledge of, and skills in, the medicines that are frequently prescribed and associated with medication-related harm. The first assessment was right after graduation, and the juniors doctors took their time (62 min (interquartile range 46–92)), so their poor performance cannot be ascribed to poor retention of knowledge and skills (which usually last about 2 years [31]) or negligence.

To contextualize the findings more broadly, this study suggests that European medical schools could benefit from implementing a final assessment focused on prescribing. Utilizing a standardized European evaluation—like the European Prescribing Exam, which is grounded in consensus studies concerning key learning outcomes, and essential medicines and diseases relevant to prescribing—could serve not only to harmonize CPT education across Europe, but also to enhance the prescribing competence of future medical professionals [13].

## Strengths and limitations

To our knowledge, this is the first longitudinal, international, and multicentre study to investigate the effect of a national prescribing safety assessment and type of curriculum on the prescribing competence of junior doctors working in clinical practice during the year after graduation. However, there are a number of limitations to take into consideration when interpreting the results. First, only one medical school used the DNPA in its curriculum at the moment we conducted this study. This uneven distribution makes it difficult to generalize the results, especially because confounders such as type of curriculum could not be tested. Second, the participants who completed the full study were possibly more interested and therefore probably more competent in CPT. This type of selection bias could have led to an overestimation of the true competence and indicates that only introducing a CPT assessment or practical learning education is not sufficient to resolve the problem of the poor prescribing knowledge and skills of junior doctors. The same could be said about the fact that the participants were not proctored during the tests. They might have used resources for the knowledge part or discussed with colleagues. Indeed, this is also true for clinical practice, where doctors can consult co-workers or formularies, but the results might be an overestimation of the true prescribing knowledge and skills. Third, the MCQs were extracted from the DNPA database. Even though all questions were different from those of the DNPA used in medical school X, the better score of this school might in part be because the junior doctors were more familiar with the type of questions and the knowledge assessed. However, the MCQs were constructed in a simple way [3], and the knowledge assessed was considered ready knowledge, i.e. all junior doctors should know it. Fourth, we could only distinguish between the type of curricula using quantitative data. Not only the type of teaching and training influence learning, but also how a teacher works, his or her ability to convey the subject manner, and the time he/she puts into it. Fifth, there are many aspects that can influence a person’s knowledge and/or skills over time, for example the ward or hospital where you work, or accessibility to continuing education. However, our international and multicentre study design probably diminished this type of bias.

## Conclusion

Optimizing and maintaining prescribing skills and knowledge from the start of a doctor’s medical career is an important step in prescribing safety. Our study shows that the inclusion of the knowledge focused Dutch National Pharmacotherapy Assessment in the medical curriculum might improve the prescribing knowledge, but not the skills, of junior doctors at the moment of graduation. However, after 6 and 12 months, both the knowledge and skills were better in the junior doctors whose medical curriculum included the DNPA. Additional studies are needed to confirm this. Moreover, a curriculum with more practical CPT education might improve prescribing knowledge and skills compared with mainly theoretical teaching. To improve the prescribing competence of future junior doctors, we recommend that a skills part is added to (national) prescribing safety assessments and that more practical teaching is incorporated in existing CPT curricula. Moreover, continuous education in CPT for junior doctors should be developed and implemented.

### Supplementary Information

Below is the link to the electronic supplementary material.

Supplementary file1 (DOCX 80 KB)

## Data Availability

The data that support the findings of this study are available directly after publication from the corresponding author upon reasonable request. Proposals may be submitted up to 24 months following the article submission. The data will be shared after de-identification.
