# Supplementary material for: The impact of a summative national prescribing assessment and curriculum type on the development of the prescribing competence of junior doctors
Source: Eur J Clin Pharmacol. 2023 Sep 22;79(12):1613–21. doi: 10.1007/s00228-023-03567-4 (PMC10663181; doi:10.1007/s00228-023-03567-4)
Supplement: Supplementary file 1 — Supplementary file1 (DOCX 80 KB) [file 228_2023_3567_MOESM1_ESM.docx]

**Supplementary Table 1. Mean knowledge score of junior doctors who graduated from medical schools that did or did not include the Dutch National Pharmacotherapy Assessment in the medical curriculum, and a mixed or theoretical curriculum**

| Assessment | With DNPA^a,b^ | Without DNPA^a,b^ | LMM | Mixed^c,d^ | Theoretical^c,d^ | LMM |
| --- | --- | --- | --- | --- | --- | --- |
| 1 | 76.7 | 67.8 | P<0.001 | 72.0 | 66.3 | P<0.001 |
| 2 | 81.8 | 76.1 | P=0.002 | 79.3 | 74.5 | P<0.001 |
| 3 | 77.0 | 70.6 | P<0.001 | 73.2 | 70.0 | P=0.026 |

DNPA: Dutch National Pharmacotherapy Assessment; LMM: Linear mixed model

^a^ In general no statistically differences in learning curve over times between the two groups (p=0.102)

^b^ More improvement for the without DNPA group in the first 6 months

^c^ In general no statistically differences in learning curve over times between the two groups (p=0.109)

^d^ More improvement for the theoretical group after one year (p=0.038)

**Supplementary Table 2. Pass rates of junior doctors who graduated from medical schools that did or did not include the Dutch National Pharmacotherapy Assessment in the medical curriculum, and a mixed or theoretical curriculum**

| Assessment | Pass | | Fail | | Chi-square |
| --- | --- | --- | --- | --- | --- |
|  | Theoretical | Mixed | Theoretical | Mixed |  |
| 1 | 13 (8.7%) | 41 (23.3%) | 137 (91.3%) | 135 (76.7%) | P<0.001 |
| 2 | 29 (19.3%) | 58 (33.0%) | 121 (80.7%) | 118 (67.0%) | P=0.006 |
| 3 | 24 (16.0%) | 40 (22.7%) | 126 (84.0%) | 136 (77.3%) | P=0.162 |
| **Overall** | **66 (14.7%)** | **139 (26.3%)** | **384 (85.3%)** | **389 (73.7%)** | **P<0.001** |

| Assessment | Pass | | Fail | | Chi-square |
| --- | --- | --- | --- | --- | --- |
|  | With DNPA | Without DNPA | With DNPA | Without DNPA |  |
| 1 | 21 (36.8%) | 33 (12.3%) | 36 (63.2%) | 236 (87.7%) | P<0.001 |
| 2 | 26 (45.6%) | 61 (22.7%) | 31 (54.4%) | 208 (77.3%) | P=0.006 |
| 3 | 16 (28.1%) | 48 (17.8%) | 41 (71.9%) | 221 (82.2%) | P=0.097 |
| **Overall** | **63 (36.8%)** | **142 (17.6%)** | **108 (63.2%)** | **665 (82.4%)** | **P<0.001** |

DNPA: Dutch National Pharmacotherapy Assessment

**Supplementary Table 3. Mean knowledge score (%) per assessment per medical school**

| Assessment | Medical school | | | | | | | | | | | ANOVA |
| --- | --- | --- | --- | --- | --- | --- | --- | --- | --- | --- | --- | --- |
|  | X | 1 | 2 | 3 | 4 | 5 | 6 | 7 | 8 | 9 | 10 |  |
| 1 | 76.7 | 76.9 | 75.0 | 69.0 | 65.3* | 63.7* | 71.9 | 65.6* | 65.4* | 61.6* | 60.6* | p<0.001 |
| 2 | 81.8 | 81.1 | 82.4 | 77.4 | 76.2 | 73.0* | 79.6 | 74.2* | 75.0 | 69.6* | 68.0* | p<0.001 |
| 3 | 77.0 | 78.0 | 71.8 | 70.8 | 68.6 | 70.9 | 75.3 | 68.9 | 70.9 | 65.1* | 65.4 | p=0.003 |

* = scored significantly worse than school X (p<0.05 in ANOVA with post-hoc Tukey’s HSD)

**Supplementary Table 4. Prevalence (%) of surgical and non-surgical doctors in the group with or without a DNPA in their curriculum**

| Group | Surgical doctors (%) | Non-surgical doctors (%) | Unknown (%) |
| --- | --- | --- | --- |
| With DNPA | 7.0 | 77.2 | 15.8 |
| Without DNPA | 11.5 | 74 | 14.5 |

DNPA: Dutch National Pharmacotherapy Assessment

**Supplementary Table 5. Prevalence (%) of (non-)registrars and research physicians in the group with or without a DNPA in their curriculum**

| Group | Registrars (%) | Non-registrars (%) | Research physicians | Unknown (%) |
| --- | --- | --- | --- | --- |
| With DNPA | 8.8 | 70.2 | 3.5 | 17.5 |
| Without DNPA | 28.6 | 41.6 | 11.5 | 18.2 |

DNPA: Dutch National Pharmacotherapy Assessment

**Supplementary Figure 1. Mean knowledge scores (%) per assessment per medical school.**

Comparison between medical school X and the other medical schools. Assessment 1: six medical schools scored significantly lower than medical school X; in assessment 2 four medical schools; in assessment 3 one medical school.

* p<0.001. Striped medical schools have mixed type curricula, non-striped theoretical curricula.

**Supplementary Figure 2. Total skills scores (%) per assessment per medical school.**

Comparison between medical school X and the other medical schools. Assessment 1: two medical schools scored significantly lower than medical school X; in assessment 2 three medical schools; in assessment 3 six medical schools university * p<0.05. Striped medical schools have mixed type curricula, non-striped theoretical curricula.
